# The variable success of in vitro maturation: can we do better?

**DOI:** 10.21451/1984-3143-AR2018-0021

**Published:** 2018-08-03

**Authors:** Alberto M. Luciano, Federica Franciosi, Rodrigo G. Barros, Cecilia Dieci, Valentina Lodde

**Affiliations:** Reproductive and Developmental Biology Laboratory, Department of Health, Animal Science and Food Safety, University of Milan, 20133 Milan, Italy

**Keywords:** cAMP, cGMP, chromatin, gap junction, meiotic arrest, oocyte, pre-IVM.

## Abstract

The efficiency of *in vitro* assisted reproductive technologies, consisting of the transfer of embryos obtained *in vitro* through *in vitro* maturation, *in vitro* fertilization and early embryo culture is still limited. The quality of the oocytes is pivotal for assisted reproductive efficiency and the maturation of the oocyte represents the first key limiting step of the *in vitro* embryo production system. At the time of removal from the antral follicles, the oocyte is still completing the final growth and differentiation steps, needed to provide the so-called developmental competence, i.e. the machinery required to sustain fertilization and embryo development. In mono-ovular species only one oocyte per cycle is available for procreation, therefore the current assisted reproduction techniques strive to overcome this natural boundary. However, the success is still limited and overall the effectiveness does not exceed the efficiency achieved in millions of years of mammalian evolution. One of the problems lies in the intrinsic heterogeneity of the oocytes that are subjected to *in vitro* maturation and in the lack of dedicated *in vitro* approaches to finalize the differentiation process. In this review we will try to overview some of the salient aspects of current practices by emphasizing the most critical and fundamental features in oocyte differentiation that should be carefully considered for improving current techniques.

## Introduction: the math of the *in vitro* embryo production (IVP) system

In cattle the efficiency of *in vitro* assisted reproductive technologies (ART), entailing of the transfer of embryos obtained through *in vitro* maturation (IVM), in vitro fertilization (IVF) and *in vitro* early embryo culture (IVC) is still limited.

**Figure 1:**
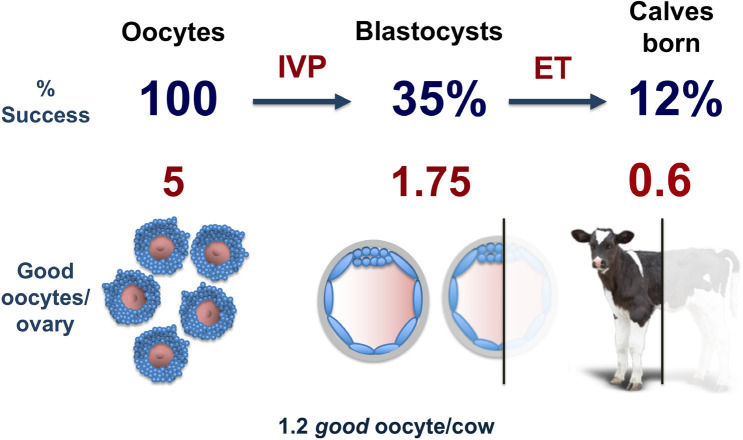
The current efficiency of standard IVP system in the bovine species.

Despite the potential advances offered by *in vitro* embryo production (IVP) systems, the percentage of success in cow remained stunning stable over the last 30 years and is limited to one third of the oocytes isolated from the ovary reaching the blastocyst stage of embryonic development (Lonergan and Fair, 2008; Galli, 2017). In bovine IVP, on a percentage basis, starting from 100 oocytes only about one third become a blastocyst after IVM, IVF and IVC, and only about one third of these embryos are able to produce a born calf (Fig. 1). Considering that, on average, 5 healthy cumulus-oocyte complexes (COCs) per ovary are collected and subjected to IVP procedures (Merton *et al.,* 2003), each ovary produces 1.75 blastocyst that finally gives approximately 0.6 calves born (Fig. 1). On a per animal basis, the IVP system brings a modest improvement respect to the physiological condition of a mono-ovular species and very close to mother’s nature results after 29 million years of natural selection and evolution in the bovine lineage.

## The physiological issue

The quality of the oocytes is pivotal for assisted reproductive technologies outcome and the maturation of the oocyte represents the first key limiting step of the IVP system. Researches are trying to deal with the improvement of IVP systems and in particular with the IVM step.

Development of IVM techniques was made possible starting from 1935’ Pincus and Enzmann observation that oocytes removed from antral follicles before natural ovulation spontaneously resume meiosis (Pincus and Enzmann, 1935). Thus, in standard IVM techniques, oocytes are collected from antral follicles and cultured *in vitro* up to the metaphase II (MII) stage with the emission of the first polar body. However, when oocytes are collected in pools from antral follicles, the processes necessary to confer full meiotic and developmental competence must be completed in a considerably high proportion of them. As a result, the oocytes ability to be fertilized or develop into embryos or to term might be compromised.

**Figure 2:**
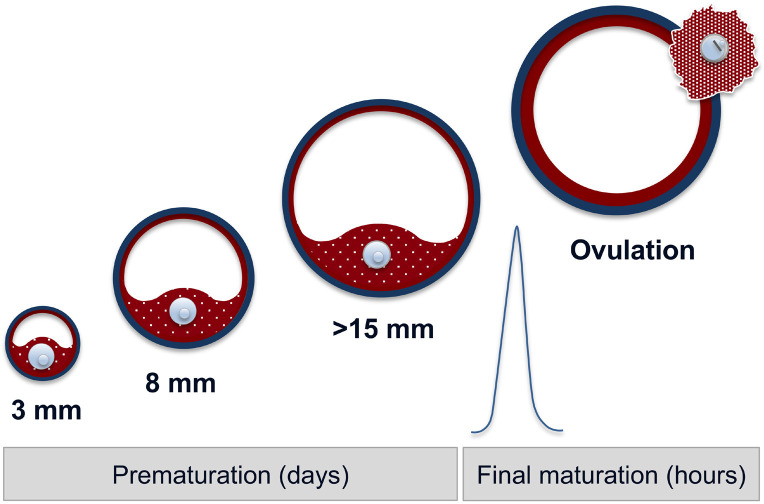
A schematic representation of the prematuration concept. Prematuration refers to the processes occurring *in vivo* during the phase of selection for dominance, when the follicles develop from 3 to 8 mm, and successively when a follicle is selected to become dominant. Finally, prematuration is accomplished shortly before the LH surge triggers the final maturation.

In the cyclic cow, the oocyte reaches its final size of around 120 µm when the follicle ranges a diameter of 2-3 mm (Fair *et al.,* 1995). The selection for dominance occurs when the follicles develop from 3 to 8 mm (Dieleman *et al.,* 2002; Adams *et al.,* 2008). Moreover, additional and essential processes occur *in vivo* during the following follicular growth and dominance phase until ovulation (Blondin *et al.,* 1997), when follicles reach a diameter of about 15 mm (Lussier *et al.,* 1987; Fortune, 1994). These processes are referred to as prematuration or capacitation and occur when a follicle is selected to become dominant and are accomplished shortly before the LH surge triggers the final maturation, as depicted in Fig. 2 (Hyttel *et al.,* 1997; Mermillod and Marchal, 1999; Dieleman *et al.,* 2002). Therefore, in the IVP practice, only few oocytes collected from 3-8 mm antral follicles develop *in vitro* into blastocysts and result in viable offspring after transfer presumably because they did not complete the dominant and preovulatory follicular development, which are crucial for the achievement of the full competence proper of the ovulatory oocyte (Hyttel *et al.,* 1997; Dieleman *et al.,* 2002).

From the above observations, it is clear that even if an oocyte collected from a 3 mm follicle has an intrinsic capacity to develop into an embryo after IVM- IVF-IVC, it still requires an additional time to acquire all of the capabilities leading to successful implantation in the uterus and maintenance of gestation to term and to yield healthy offspring, after IVF and IVC. These studies have naturally led to the design of *in vitro* approaches that take into account the need for the oocyte to complete its differentiation, namely a prematuration or capacitation step before *in vitro* maturation.

## The *in vitro* prematuration step

### Controlling cAMP

The identification of the mechanisms that control oocyte meiotic arrest and resumption (reviewed in Conti *et al.,* 2012) has provided the molecular tools to block or delay spontaneous meiotic resumption after removal from the follicle in *in vitro* prematuration studies (reviewed in Bilodeau-Goeseels, 2012). A firmly established concept is that meiotic arrest of the oocyte relies on high concentrations of the second messenger cyclic AMP (cAMP; Cho *et al.,* 1974; Dekel and Beers 1978; reviewed in Conti *et al.,* 2012), which is known to regulate oocyte and granulosa cell functions (Luciano and Peluso 1995; Conti, 2002). Oocyte cAMP level is sustained by endogenous adenylate cyclase and constitutively active G-protein-coupled receptors (Mehlmann *et al.,* 2002). cAMP is generated also by cumulus cells and then transported into the oocyte through gap junctions (Anderson and Albertini, 1976; Bornslaeger and Schultz, 1985; Luciano *et al.,* 2005). At the same time, intra-oocyte cAMP concentration is regulated by the activity of the oocyte specific phosphodiesterase 3A (PDE3A) enzyme that degrades cAMP into 5’AMP (Tsafriri *et al.,* 1996), whose activity is inhibited by cyclic guanosine 3’,5’-monophosphate (cGMP; Norris *et al.,* 2009; Vaccari *et al.,* 2009). cGMP is produced in the cumulus cells upon the activation of the guanilyl-cyclase coupled natriuretic peptide receptor type-2 (NPR2; Robinson *et al.,* 2012) whose activity is induced by its ligand natriuretic peptide precursor C (CNP), which is mainly synthesized by mural granulosa cells (Zhang *et al.,* 2010, 2011). cGMP is then transferred via gap junctions (Richard and Baltz, 2014) to the oocyte where it inhibits PDE3A thus contributing to the maintenance of meiotic arrest (Norris *et al.,* 2009; Vaccari *et al.,* 2009).

Several physiological methods for artificially maintaining meiotic arrest in bovine oocytes have been developed since 1980’ with variable success (Leibfried and First, 1980; Sirard and Bilodeau, 1990; Sirard and Coenen, 1993; De Loos *et al.,* 1994; Richard and Sirard, 1996; Fouladi Nashta *et al.,* 1998). Starting from the modulation of cAMP content, numerous IVM systems have been suggested for stimulating oocyte maturation and embryonic developmental competence acquisition (Gilchrist *et al.,* 2016).

Numerous pharmacological and physiological agents have been used to modulate the oocyte cAMP concentration in order to temporally control the oocyte’s meiotic arrest and resumption (Gilchrist *et al.,* 2016). For instance, cAMP concentration has been manipulated through the use of broad-spectrum such as IBMX or specific inhibitors of phosphodiesterases (PDEs) such as cilostamide, milrinone or Org9935, by activators of adenylate cyclase (forskolin, iAC), through cAMP analogs (dbcAMP) or by a combination of these agents. Several laboratories have shown that a delay of meiotic resumption has a beneficial effect on embryonic developmental competence. Specifically, the manipulation of intracellular cAMP concentration affects the functional coupling between oocyte and cumulus cells so that a decrease in cAMP determines a drop in gap junction-mediated intercellular communications (Luciano *et al.,* 2004; Thomas *et al.,* 2004). On the other hand, treatments that sustained the intracellular cAMP level prevented the loss of cumulus- oocyte communications and increased oocyte developmental competence (Luciano *et al.,* 1999, 2011; Guixue *et al.,* 2001; Modina *et al.,* 2001; Atef *et al.,* 2005; Nogueira *et al.,* 2006; Ozawa *et al.,* 2008; Shu *et al.,* 2008; Nogueira and Vanhoutte, 2009; Albuz *et al.,* 2010; Dieci *et al.,* 2013; Lodde *et al.,* 2013; Rose *et al.,* 2013; Zeng *et al.,* 2013; Richani *et al.,* 2014). In different systems the preservation of the cAMP concentration during several hours of prematuration culture seems to be the main requirement to promote regular chromatin transition thus endorsing oocyte differentiation (Nogueira *et al.,* 2006; Vanhoutte *et al.,* 2007; Luciano et al., 2011; Dieci *et al.,* 2013; Lodde *et al.,* 2013; Sanchez *et al.,* 2015) and an increase in embryonic developmental competence and/or quality (Luciano *et al.,* 2011; Dieci *et al.,* 2013; Lodde *et al.,* 2013; Franciosi *et al.,* 2014; Azari-Dolatabad *et al.,* 2016; Li *et al.,* 2016; Park *et al.,* 2016; Soares *et al.,* 2017).

However, in most of the above cited cases the reported results showed only a slight improvement in developmental competence depending on the approach used. Often, rather than increasing the number of blastocysts obtained, an improvement in the parameters related to embryo quality was observed (Luciano *et al.,* 2011; Dieci *et al.,* 2013, 2016; Lodde *et al.,* 2013; Rose *et al.,* 2013; Franciosi *et al.,* 2014; Zeng *et al.,* 2014; Azari-Dolatabad *et al.,* 2016; Li *et al.,* 2016; Park *et al.,* 2016; Sanchez *et al.,* 2017; Soares *et al.,* 2017). For instance, blastocysts with higher number of cells or embryo with better developmental kinetics. In other cases, results showed no significant improvement over the current standard *in vitro* embryo production system (Jee *et al.,* 2009; Gharibi *et al.,* 2013; Guimaraes *et al.,* 2015; Diogenes *et al.,* 2017).

### Controlling MPF

In addition to cyclic nucleotide manipulation- based methods, other approaches have been used to prevent meiotic resumption. Downstream to cAMP transduction pathway is M-phase promoting factor (MPF), a heterodimer consisting of a kinase, cdk1 and its regulatory partner, cyclin B (cdk1-cyclin B), which is involved in the regulation of G2/M cell cycle transition of all eukaryotic cells. Cyclic AMP-mediated Protein Kinase A (PKA) activity inhibits Cdk1 hence contributing to oocyte meiotic arrest (Mehlmann, 2005). The activation of MPF is also a key point of meiotic resumption in oocytes that corresponds to a G2/M transition (Jones, 2004; Mehlmann, 2005).

Several pharmacological approaches aimed to interfere with MPF activity have been used to artificially maintain mammalian oocyte in meiotic arrest. Cell permeable and selective inhibitors of the CDK1/cyclin B kinase, butyrolactone-I (Kitagawa *et al.,* 1993) and roscovitine (Meijer *et al.,* 1997) have received more attention. Butyrolactone-I has been shown to reversibly inhibit meiotic resumption in bovine (Kubelka *et al.,* 2000; Lonergan *et al.,* 2000; Ponderato *et al.,* 2001, 2002; Imai *et al.,* 2002; Adona *et al.,* 2008; Ferreira *et al.,* 2009; De Bem *et al.,* 2011) and pig (Wu *et al.,* 2002) oocytes for 24 h without negatively affecting the subsequent development to the blastocyst stage. Similarly, roscovitine was effective in reversibly maintain oocyte meiotic arrest in both cow (Mermillod *et al.,* 2000; Sa Barretto *et al.,* 2011) and pig (Marchal *et al.,* 2001). Moreover, the combination of both substances did not cause detrimental effects on development to the blastocyst stage (Ponderato *et al.,* 2001), and subsequent early stages of organogenesis (Ponderato *et al.,* 2002). However, when compared to the standard IVP system, the majority of the studies reported no significant improvements in embryonic developmental competence when oocytes were arrested with MPF inhibitors and subsequently matured and fertilized *in vitro*. Nonetheless, the use of butyrolactone and roscovitine have been reported to induce some modifications in the oocytes at ultrastructural level (Lonergan *et al.,* 2003), and whether or not these modifications are compatible with normal gestation and live births is still debated.

## What’s the matter with *in vitro* prematuration?

Notwithstanding the meiotic arrest method used, little to no improvement has been observed in the embryonic developmental competence when oocytes were cultured in bulk, regardless their follicular origin. We believe that the high heterogeneity of the population of COCs subjected to *in vitro* prematuration protocols is responsible for its limited success rate. In most of the cases, indeed, studies on *in vitro* prematuration efficiency have been conducted on ovaries obtained from slaughtered animals, thus contain all types and sizes of antral follicles representing different stages of oocyte development. Considering that in the absence of hormonal synchronization treatments, the follicle population in the ovary is heterogeneous, the stage of differentiation of the oocyte should be taken into account (Luciano and Sirard, 2018). In the following paragraphs we will describe the studies that sustain this hypothesis and, at the same time, have led to define morphological and molecular markers to identify the most suitable prematuration conditions.

## A pre-maturation approach thoughtful of oocyte physiology

The oocyte acquires developmental competence just prior to ovulation. Oocyte developmental competence is usually defined as the ability of a female gamete to mature into an egg with its capability to be fertilized and sustain embryo development to the blastocyst stage (Conti and Franciosi, 2018). Nevertheless, identifying oocytes that have achieved the final competence is extremely arduous. One morphological indicator is large-scale chromatin configuration, which changes while the oocyte grows and differentiates during follicular antral development (De La Fuente, 2006; Luciano and Lodde, 2013). This has led to the identification of distinct stages in which the chromatin becomes progressively more compact and occupies a smaller area of the oocyte nucleus or germinal vesicle (GV) in all mammals studied so far (reviewed in Luciano and Lodde 2013; Luciano *et al.,* 2014).

In cows, the four chromatin configurations described correspond to different stages of developmental competence (Lodde *et al.,* 2007). In the GV0 configuration the chromatin appears mostly uncondensed and dispersed throughout the nucleoplasm, while the appearance of few foci of condensation marks the transition to the GV1 configuration. Further compaction into distinct aggregates characterizes the GV2 configuration while the highest level of compaction occurs in GV3, where the chromatin appears as a single clump in a restricted area of the nucleus (Lodde *et al.,* 2007). These stages accompany follicle development. Nearly 90% of the oocytes isolated from early antral follicles (0.5 to 2 mm in diameter) show a GV0 configuration, while medium antral follicles (2-8 mm), which are the follicles most commonly used for IVP, contain nearly no GV0-stage oocytes but GV1, GV2 and GV3 stages in similar proportions (Lodde *et al.,* 2007).

Chromatin configuration is not simply morphology, but a marker of gamete differentiation associated with various functional features (Luciano *et al.,* 2014). In bovine oocytes, the transition from GV0 to GV3 corresponds to progressive transcription silencing (Lodde *et al.,* 2008), changes in epigenetic signatures such as overall methylation (Lodde *et al.,* 2009) and histone modification (Labrecque *et al.,* 2015; Lodde *et al.,* 2017) and changes in nuclear architecture and cytoplasmic organelle redistribution (Lodde *et al.,* 2008). More importantly, the transition from dispersed to compacted chromatin is accompanied by gradual acquisition of meiotic and developmental competence (Lodde *et al.,* 2007, 2008; Luciano *et al.,* 2011). Similar correlations have been described in mice and humans (Zuccotti *et al.,* 1995; Bouniol-Baly *et al.,* 1999; Combelles *et al.,* 2003; Miyara *et al.,* 2003; Sanchez *et al.,* 2015). It is noteworthy that the changes in chromatin configuration also accompany significant changes in the transcriptome signature in the oocyte (Labrecque *et al.,* 2015) and in the corresponding cumulus cells (Dieci *et al.,* 2016) suggesting that chromatin configuration also reflects phases of follicle development (Luciano and Sirard, 2018). Moreover, large-scale changes in chromatin configuration are related to gap-junction functional status through cAMP dependent mechanisms (Luciano *et al.,* 2011; Lodde *et al.,* 2013;Franciosi *et al.,* 2014). In cumulus-oocyte complexes isolated from early antral follicles, characterized by a GV0 chromatin configuration, the maintenance of functional gap-junction communications promotes oocyte growth, gradual transcriptional silencing, large-scale chromatin remodeling and competence acquisition, all of which are controlled via cAMP mediated mechanism (Luciano *et al.,* 2011).

**Figure 3:**
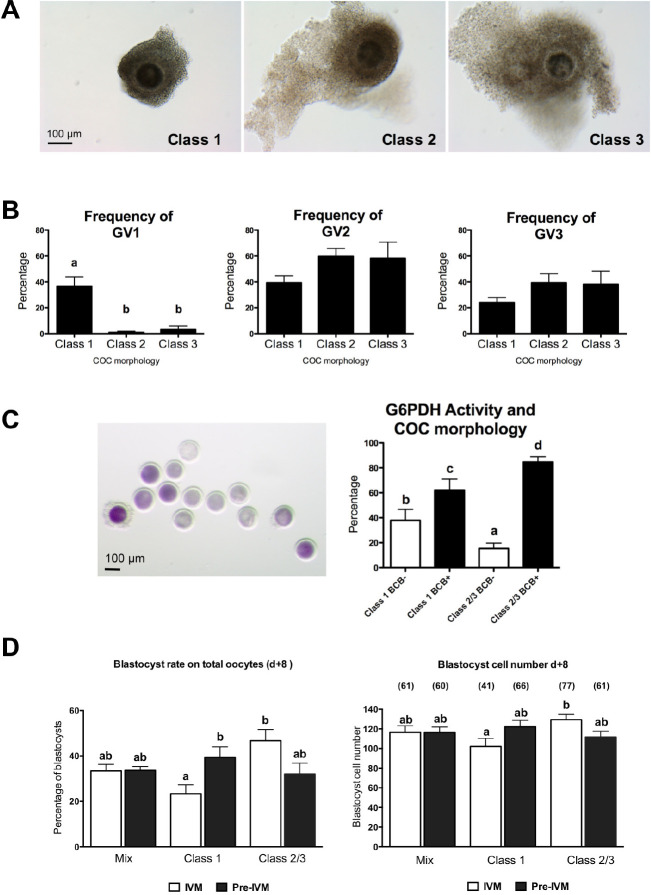
A. Representative images of class 1, class 2 and class 3 COCs. (Class 1: homogeneous ooplasm and absence of expansion of outer layer CC; Class 2: minor granulation of the ooplasm and/or beginning of expansion of outer layer CC; Class 3: highly granulated ooplasm and few CC layers showing expansion). B. Graphs show the frequency of GV1, GV2 and GV3 chromatin configurations in each class. C. After collection, COC were separated into Class 1 and Class 2/3 on the basis of their morphology and subjected to Brilliant Cresyl Blue staining (BCB). After removal of CC, oocytes were classified as BCB+ or BCB- as shown in the representative picture. Graph shows the percentage of BCB+ and BCB- oocytes in Class 1 and 2/3 COC. A total of 337 COC were analyzed (126 Class 1 and 211 of Class 2/3) in nine independent experiments. D. Effect of pre-maturation treatment on COC with different morphology. After collection, COC were separated into Class 1 and Class 2/3 on the basis of their morphology and *in vitro* matured with or without the pre-IVM treatment. Then, oocytes were in-vitro fertilized and in-vitro cultured for 8 days. Groups of unsorted COC (mix of Class 1/2/3) were subjected to the same experimental procedure and were used as controls. Graphs show the effect of the pre-IVM treatment on the blastocyst rate (left) and mean cell number per blastocyst (right). A total of 947 oocytes were analyzed in this study (292 mixed oocytes, 321 Class 1 and 334 Class 2/3) in six independent experiments.

A clear indication that the success of *in vitro* prematuration using cAMP modulators is affected by oocyte heterogeneity, comes from our recent study in cows showing that prematuration may be beneficial for the developmental competence of GV1 oocytes but detrimental for that of GV3 oocytes (Dieci *et al.,* 2016). Previous studies have shown that oocytes within COCs with compact cumulus and homogeneous ooplasm are less competent than those in which the ooplasm appears granulated and the outer layers of cumulus cells exhibit the slight expansion often seen in early atretic follicles (Blondin and Sirard, 1995). In our study, we have shown that oocytes within COCs with compact cumulus and homogeneous ooplasm, tend to be in the GV1 chromatin configuration (loosely condensed). On the contrary in COCs with slight expansion and/or granulated cytoplasm the oocyte chromatin is in either the GV2 or the GV3 configuration, while GV1 representation is negligible (Fig. 3 A, B; Dieci *et al.,* 2016). In our experiments the GV1-enriched oocyte population benefited of a 6 h of prematuration treatment with cilostamide and physiological concentration of FSH, whereas standard IVM without pretreatment leads to poor pre-implantation development. Strikingly, the same prematuration protocol decreases the blastocyst rate of GV2-GV3 enriched oocyte population (Dieci *et al.,* 2016), which performed better if directly processed for IVM (Fig. 3 D).

Data were analyzed by one-way ANOVA followed by Newman-Keuls multiple comparison test; data are expressed as means ± SEM; a, b: different letters indicates significant differences (P < 0.05). From Dieci *et al*., 2016.

Noteworthy, in animal models characterized by a more homogeneous population of oocytes, prematuration protocols substantially improved the developmental competence. Significant effects were obtained in oocytes with an inherent low embryonic developmental competence such as prepubertal calves, with the use of butyrolactone-I and roscovitine (Donnay *et al.,* 2004) and juvenile mice, with the use of CNP and low concentration of FSH (Romero *et al.,* 2016). Oocytes isolated from early antral follicles (mostly GV0), growth in the presence of cilostamide and a physiologic concentration of FSH were successfully brought to meiotic and embryonic developmental competence (Luciano *et al.,* 2011). In the same line, human oocytes derived from small antral follicles, which have an intrinsically low developmental potential (Sanchez *et al.,* 2017), or compact COCs that are less meiotically competent and characterized by uncondensed chromatin (Nogueira *et al.,* 2006; Sanchez *et al.,* 2017), progressed to a condensed chromatin configuration when prematuration was applied (Vanhoutte *et al.,* 2007).

Moreover, when COCs were selected as homogeneous populations of growing and fully grown oocytes by brilliant cresyl blue staining (Fig. 2 C), the population of growing oocytes was greatly enhanced in embryonic developmental capability while the effect of prematuration was detrimental on the population of fully grown oocytes (Azari-Dolatabad *et al.,* 2016; Dieci *et al.,* 2016; Wang *et al.,* 2016).

## Conclusions

The goal of IVP technologies is to improve oocyte quality through a physiological approach in order to obtain a higher number of oocytes with elevated developmental competence. Oocytes coming from non-ovulatory follicles, although can spontaneously resume meiosis are still far to be fully competent. Regardless of the meiotic arrest method used, the rationale of most of the studies aimed at reproducing *in vitro* the final stages of prematuration that normally occur *in vivo*. Several studies have served as proof of concept that prematuration can improve the developmental competence of the oocyte. Nevertheless, little advance has been observed in the embryonic developmental competence when oocytes were cultured in bulk regardless their follicular origin.

The heterogeneity of oocyte population at the start of the procedure greatly affects the outcome of prematuration systems and the specific metabolic needs of the oocyte at the time of isolation should be taken into account. The identification of specific non-invasive biomarker(s) of oocyte health status and final differentiation can provide useful tools for the selection of good quality oocyte, time of prematuration (culture length) as well as the specific environment (hormones, growth factors, molecules, etc.) for optimizing prematuration culture systems. At the same time, the definition of customized culture system can be associated with stimulation strategies to synchronize the growth of ovarian follicles in the donor in order to obtain oocytes specifically suitable for tailored prematuration protocols.

## Competing interest

The authors declare that there is no conflict of interest that could be perceived as prejudicing the impartiality their presentation of the research findings mentioned in this work.
